# Patient and Provider Demographics and the Management of Genitourinary Tract Infections in the Emergency Department

**DOI:** 10.1155/2023/1522347

**Published:** 2023-09-11

**Authors:** Johnathan M. Sheele, Lanyu Mi, Jessica Monas, Michael Mohseni

**Affiliations:** ^1^Mayo Clinic, Department of Emergency Medicine, Jacksonville, FL 32224, USA; ^2^Mayo Clinic, Division of Clinical Trials and Biostatistics, Department of Quantitative Health Sciences, Scottsdale, AZ, USA; ^3^Mayo Clinic, Department of Emergency Medicine, Phoenix, AZ, USA

## Abstract

**Introduction:**

Urinary tract infections (UTIs) and sexually transmitted infections (STIs) can have overlapping signs, symptoms, and findings on urinalysis. Our objective was to determine if patient or provider demographics are associated with differences in the diagnosis and management of UTIs and STIs in the emergency department (ED).

**Methods:**

We analyzed 38,062 ED patient encounters from a single healthcare system between April 18, 2014, and March 7, 2017. All encounters were women ≥18 years of age and not admitted to the hospital. We performed logistic regression using patient and provider demographics, laboratory testing results, ED triage data, and ED diagnoses.

**Results:**

The patient's age, race, and marital status were not associated with having an ED UTI diagnosis with a urine culture ≥10,000 colony forming units (CFUs)/mL (vs. <10,000 CFUs/mL). Patient race and the sex of the ED provider were not associated with differences in empiric antibiotic treatment for gonorrhea and chlamydia during the ED encounter. Patient's race and the sex of the ED provider were also not associated with discordance between empiric antibiotic therapy given in the ED and the results of gonorrhea and chlamydia tests that resulted following the ED encounter.

**Conclusion:**

In our multivariate analyses, we did not observe that the patient's race resulted in significant differences in the diagnosis of UTIs with bacteriuria ≥10,000 CFU/mL or differences in the empiric treatment of gonorrhea and chlamydia infections among those tested for the infection in the ED. The patient's age and marital status, but not the provider's sex, were significantly associated with differences in the management of gonorrhea and chlamydia.

## 1. Introduction

Rates of gonorrhea and chlamydia are rising in the United States, and an increasing number of sexually transmitted infections (STIs) are being diagnosed in the emergency department (ED) [1–4]. Approximately, 20% of Americans have an STI and new STIs result in $16 billion in direct medical care [5]. Managing STIs in the ED can be challenging because patients can be asymptomatic or present with varied signs and symptoms. STIs in women can be challenging to differentiate clinically from urinary tract infections as both can have overlapping signs, symptoms, and laboratory findings on urinalysis. Furthermore, the results of nucleic acid amplification tests (NAATs) for gonorrhea, chlamydia, and trichomonas may not be available to the clinician during the patient's ED encounter requiring providers to either empirically treat for STIs in the ED or wait for the results of diagnostic testing. If a woman goes untreated for an STI, the risks may include chronic pelvic pain, infertility, genital cancers, and an increased risk for HIV [2]. These risks must be balanced against the harms of administering unnecessary antibiotics.

Urinary tract infections (UTIs) are among the most common bacterial infections diagnosed in the emergency department (ED) [6]. An estimated one fifth of ambulatory medicine encounters involving UTI occurs in the ED [6]. The diagnosis of a bacterial UTI traditionally relies on the patient's signs, symptoms, or both, of a UTI plus a positive urine culture. However, urine culture results are unavailable during the clinical encounter, and ED clinicians are frequently noncompliant with evidence-based recommendations for UTI management [7]. Not all ED patients diagnosed with a UTI receive a urine culture. Patients at risk for sexually transmitted infections (STIs) and with concurrent genitourinary complaints do not always receive ED testing for STIs [8]. As a result, ED clinicians likely overdiagnose UTIs and underdiagnose STIs [8].

Racism, racial disparities, and implicit bias exist within our healthcare system, adversely affecting the quality of care [9, 10]. Previous studies have shown racial inequality in STI testing, although there are few studies related to STIs in the ED [11–15]. Little has been published on disparities in diagnosing and managing UTIs, particularly in the ED [16–18]. Our primary study objective was to determine if a patient's race is associated with differences in the diagnosis and management of genitourinary tract infections. Secondarily, we looked at other patient and provider demographics to determine if they were also associated with differences in the diagnosis and management of STIs and UTIs. Specifically, we looked for differences in bacteriuria in the urine culture among women diagnosed with a UTI, the empiric treatment of gonorrhea and chlamydia during the ED encounter, and the frequency with which there was discordance with gonorrhea and chlamydia test results and the administration of empiric antibiotic for those infections during the ED encounter.

## 2. Materials and Methods

The <removed for blinding> institutional review board approved the study with a waiver of informed consent. We utilized an existing data set of 75,000 ED patient encounters, but our analysis only included 38,254 women who were not admitted to the hospital. All patient encounters in the data set were at least 18 years of age and seen at university hospitals between April 18, 2014, and March 7, 2017. All patient encounters in the data set were tested for gonorrhea, chlamydia, or trichomonas or they had a urinalysis plus urine culture during their ED visit. Patients who received empiric antibiotics for STIs but did not receive STI testing were not included in the data set. Similarly, the data set did not include patients who received a urinalysis without a urine culture or STI testing. The data set contained retrospective data extracted from the electronic medical records by an institutional information technology team. Data from this data set have been published previously, including how the data set was created [19–28].

Women who received azithromycin and ceftriaxone in the ED or ceftriaxone in the ED, plus an outpatient prescription for doxycycline, were considered to be treated for gonorrhea and chlamydia. These were the management recommendations for gonorrhea and chlamydia when clinical care was administered [29]. Women not receiving those antibiotics during their ED encounter were considered not empirically treated for gonorrhea and chlamydia. We considered ED testing and treatment for gonorrhea and chlamydia to be concordant when (1) a patient was empirically treated for gonorrhea and chlamydia and had a positive NAAT for gonorrhea, chlamydia, or both or (2) a patient was not empirically treated for gonorrhea and chlamydia and they had both negative gonorrhea and chlamydia NAATs. We considered ED testing and treatment for gonorrhea and chlamydia to be discordant when (1) a patient was empirically treated for gonorrhea and chlamydia, but they were negative for both gonorrhea and chlamydia by NAAT or (2) a patient was not empirically treated for gonorrhea and chlamydia but had a positive NAAT for gonorrhea, chlamydia, or both. Indeterminant or erroneous NAAT results for gonorrhea and chlamydia were not included in the analysis.

A urinalysis was performed if any test from the urinalysis was reported. The urine source was categorized as clean catch/voided, “other” (i.e., bladder catheter, straight catheter, ileostomy, nephrostomy, suprapubic, or urostomy, etc.), or it was not documented. The urinalysis was categorized as follows: bacteria (0–4+), blood (0–3+), glucose (positive or negative), ketones (positive or negative), leukocyte esterase level (0–3+), mucous (0–4+), nitrite (positive or negative), pH [5–9], protein (positive or negative), red blood cells (RBCs) 0–101 cells/high powered field (cells/HPF), trichomonas (positive or not reported), white blood cell (WBC) clumps (positive or negative), WBCs 0–101 (cells/high powered field (HPF)), and yeast (present or none). The clinical laboratories reported urine RBCs and WBCs in various ways. The mean was used in the analysis if a range was reported. If >100 cells/HPF were reported, 101 cells/HPF were used for analysis. The urine culture was classified as ≥10,000 colony forming units (CFUs)/mL or <10,000 CFUs/mL. All laboratory testing and cultures were ordered at the treating clinician's discretion.

If any wet mount results were reported, women were categorized as having a vaginal wet mount. Vaginal wet mount clue cells, trichomonas, and yeast were either present or absent. Vaginal wet mount WBCs were categorized as ≤10 or 11–100 cells/HPF [24].

There were 1,913 women who tested positive for *T. vaginalis*, and this was by urinalysis 384/26,880 (1.4%), vaginal wet mount 1,354/15,572 (8.7%), and NAAT 418/4,923 encounters (8.5%); however, the women may have had more than 1 positive test during the encounter. ED clinicians do not use the urinalysis to diagnose *T. vaginalis* because of low sensitivity (but does have high specificity). The vaginal wet mount has moderate sensitivity and high specificity, while the NAAT has high sensitivity and specificity [30–32]. Both the urine microscopy and vaginal wet mount results are available to the clinician during the ED encounter, and to avoid multicollinearity in the multivariable analysis, we combined these *T. vaginalis* results into a single variable, indicating whether *T. vaginalis* infection was known during the ED encounter as follows: (1) positive for *T. vaginalis* during the ED encounter: positive result by urine microscopy, vaginal wet mount, or both; (2) negative for *T. vaginalis* infection during ED encounter: negative *T. vaginalis* by vaginal wet mount and negative by urine microscopy if the urinalysis was performed; and (3) vaginal wet mount not performed: vaginal wet mount not performed and urine microscopy negative for *T. vaginalis* if a urinalysis was performed. The *T. vaginalis*, *N. gonorrhea*, and *C. trachomatous* NAAT Aptima (Hologic) were considered separately because these test results did not result until after the ED visit. When examining women empirically treated for gonorrhea and chlamydia in the ED and for concordance and discordance between empiric antibiotics for gonorrhea and chlamydia during the ED encounter and the testing results, we only examined women who had both a positive or negative NAAT for gonorrhea and chlamydia.

The demographic variables used in the analysis included the patient's age (18–28, 29–39, 40–50, 51–61, and ≥62 years), race (Black/African American (*n* = 25,429), White (11,995), and Asian (*n* = 163) which we combined with “other” (*n* = 475) in our analysis), and marital status. We considered age a categorical variable given the nonlinear distribution of STIs across the age spectrum [27, 29]. There were 192 persons of unknown race and 131 persons with the unknown marital status. The sex and training level of the primary clinician who was principally caring for the patient were considered in our multivariable modeling as follows: (1) the resident: for any patient encounter involving a resident; (2) the attending: for any attending physician-only encounters; and (3) the advanced practice practitioner (APP): for any encounter involving an APP or an APP plus an attending physician. The principal clinician caring for the patient was considered to be a resident for encounters involving a resident, APP, and an attending physician because residents would occasionally sign out unfinished patients to an APP but APPs would not sign out cases to residents. Both APPs and residents would occasionally sign out unfinished patient encounters to attending physician or to another APP or resident, respectively.

The following triage and encounter data were included in the analysis if it was present for the clinical encounter: whether the patient has a primary care physician documented, the method of ED arrival, and the triage emergency severity index (ESI). Women were diagnosed with a UTI if they had an International Classification of Diseases (ICDs), Ninth Revision or Tenth Revision, code of N30.90, O86.22, N30.00, N30.91, N30, N30.0, N30.01, N30.9, O23.10, O86.20, N39.0, O08.83, O03.38, O04.88, O03.88, O86.2, O86.29, O23.40, 646.64, 599.0, 639.8, 646.60, 595.0, 595.9, or 595.89. Women were considered pregnant if they had a documented positive pregnancy test or an ICD Ninth Revision or Tenth Revision, code of O00.1, O00.8, O21.9, O00.90, Z32.01, O00, O20, V72.42, 643.93, or 633.90.

### 2.1. Statistical Analysis

Patient demographics, clinical characteristics, and provider characteristics were summarized as count (percentage) for categorical variables and median (interquartile range) for continuous variables. Univariate and multivariable logistic regression analyses were used to investigate the associations between our outcomes of interest and patient/provider characteristics. Variables for the regression analysis were chosen based on available patient and provider data (Table 1). Not all women undergoing STI testing had a urinalysis or a vaginal wet mount. However, we included these variables in our regression analyses because genitourinary tract infections can cause nonspecific inflammatory changes on both tests, which could have influenced clinician management decisions [8]. The analyses were conducted by BlueSky Statistics software v7.40 (BlueSky Statistics LLC). All tests were two sided, and *p* values <0.05 were considered significant.

## 3. Results

38,254/75,000 (51%) encounters in the data set met our inclusion criteria of being a woman who was not admitted to the hospital and were included in the analysis. Descriptive statistics are summarized in Table 1. Within our cohort, UTI (*n* = 8,330) was diagnosed in 1,437 (17.3%) women with no urine culture performed, 3,915 (47.0%) with <10,000 CFU/mL bacterial growth, and 2,978 (35.8%) with ≥10,000 CFU/mL bacterial growth. 6,893 (82.7%) women diagnosed with a UTI received a urine culture. A total of 22,668 women received a urine culture, of which 5,655 (24.9%) had ≥10,000 CFU/mL bacterial growth. Among those women with ≥10,000 CFU/mL bacterial growth on urine culture, 2,978 (52.7%) were diagnosed with a UTI.

Among those being diagnosed with a UTI in the ED and with a documented patient race, they were Black/African American (*n* = 4,534), White (*n* = 3,611), and Asian/others (*n* = 142) (Table 2). The rates of being diagnosed with a UTI and having a urine culture ≥10,000 CFU/mL were 1552 (34.3%) for Black/African American, 1355 (37.5%) for White, and 53 (37.3%) for Asian/others.

There were 16,317 patient encounters with positive or negative test results for gonorrhea, chlamydia, or both. Among these, they were Black/African American (*n* = 14,522; 89.0%), White (1,509; 9.2%), Asian/others (*n* = 217; 1.3%), and unknown race (69; 4.2%). Their marital status was reported as married/life partner (*n* = 1,432; 8.8%), single (14,210; 87.1%), widowed (72; 0.4%), divorced/separated (556; 3.4%), and unknown marital status 47 (0.3%). There were 1,300 (8.0%) who tested positive for chlamydia, 473 (2.9%) who tested positive for gonorrhea, and 149 (0.9%) who tested positive for both gonorrhea and chlamydia. There were 1,516/14,481 (10.5%) tested Black/African American women infected with gonorrhea, chlamydia, or both compared with 90/1,503 (6.0%) of White women (*p* < 0.001).

There were 16,199 encounters with both NAAT results being positive or negative for gonorrhea and chlamydia (Table 3). Of these, 3,068 (18.9%) were given empiric antibiotics for gonorrhea and chlamydia during the ED encounter compared to 13,131 (81.1%) not given empiric antibiotics for gonorrhea and chlamydia. Women had concordance with being given empiric antibiotics in the ED for gonorrhea and chlamydia and their NAAT results for one or both of those infections (*n* = 12,526; 77.3%) compared with discordance between empiric antibiotics in the ED and their NAAT result (*n* = 3,673; 22.7%).

### 3.1. UTI Diagnosis and ≥10,000 CFU/mL Bacteriuria vs. <10,000 CFU/mL Bacteriuria on Urine Culture

Our univariate analysis is available as Supplementary file 1. The patient's race was not associated with a UTI diagnosis and having a urine culture grow ≥10,000 CFU/mL bacterial growth compared to being diagnosed with a UTI and having <10,000 CFU/mL (Table 4). The following were associated with being diagnosed with a UTI and having a urine culture ≥10,000 CFU/mL bacterial growth when compared to being diagnosed with a UTI and having <10,000 CFU/mL bacterial growth: ESI 4-5 (vs. 1-2); the patient was seen by a resident and attending (vs. an APP); urine source was clean catch/voided and “other” (vs. not documented); on urinalysis: higher urine bacteria, more blood, fewer ketones, less mucus, nitrite positive, more WBC clumps, more urine WBCs, and on vaginal wet mount: having ≤10 WBCs/HPF (vs. 11–100 WBCs/HPF); negative *T. vaginalis* NAAT (vs. no test); negative for *T. vaginalis* infection during ED encounter (vs. positive for *T. vaginalis* during the ED encounter); and a negative *T. vaginalis* NAAT (vs. no test) (vs. 11–100 cells/HPF) (*p* < 0.03 for all) (Table 4).

### 3.2. Empiric Treatment for Gonorrhea and Chlamydia in Those Tested for the Diseases

Our univariate analysis is available as Supplementary file 2. The following were significantly associated with being empirically treated for gonorrhea and chlamydia in the ED (vs. not being empirically treated in the ED): age 18–28, 29–39, 40–50, and 51–61 years (vs. ≥62 years); separated/divorced (vs. married/life partner); single (vs. married/life partner); not being pregnant (vs. pregnant); APP (vs. attending only); urine source clean catch/voided (vs. not documented); on urinalysis: less blood urine, higher leukocyte esterase, lower RBCs, and no yeast present (vs. present); and on vaginal wet mount: clue cells negative (vs. not performed), WBC 11–100 cells/HPF (vs. <10 cells/HPF); on NAAT: no gonorrhea test result and a positive result (vs. negative), trichomonas positive (vs. negative); not diagnosed with a UTI in the ED; and positive for *T. vaginalis* during the ED encounter (vs. negative for *T. vaginalis* infection during ED encounter) (*p* < 0.05 for all) (Table 5). The patient's race and the sex of the primary ED provider were both not significant.

### 3.3. Discordance between Testing Results for Gonorrhea and Chlamydia and Empiric Treatment in the ED

The following were significantly associated with being empirically treated for gonorrhea and chlamydia in the ED if testing negative or not being treated for gonorrhea or chlamydia and testing positive for those infections (vs. not being empirically treated for gonorrhea and chlamydia in the ED if testing negative or empirically treated for gonorrhea or chlamydia and testing positive for those infections): age ≥62 years (vs. 18–28, 29–39, 40–50, and 51–61 years); married/life partner (vs. separated/divorced); married/life partner (vs. single); pregnant (vs. not); urine source not documented (vs. clean catch/voided); on urinalysis: higher blood, lower leukocyte esterase, and yeast present (vs. not); urine culture <10,000 CFU/mL (vs. not urine culture done); on vaginal wet mount: clue cell negative (vs. positive), WBC ≤10 WBC/HPF (vs. 11–100 WBC/HPF), yeast preset (vs. absent); and *T. vaginalis* NAAT negative (vs. positive) (*p* < 0.02 for all) (Table 6). Neither the patient's race, sex of the primary ED provider, nor the training level of the primary ED provider was associated with our outcome (Table 6).

## 4. Discussion

Our study examined whether the patient's race was associated with differences in diagnosing and managing genitourinary tract infections. We found that patient's race was not associated with differences in bacteriuria on urine culture among women diagnosed with a UTI. Our findings concord with a survey of implicit bias among pediatricians and found no significant relationships for treating UTI in Black patients [10]. Another study found no significant differences between ED patients receiving inappropriate treatment for UTIs when examining age, patient's race, and the professional level of training by the treating clinician [7].

We also found that patient's race was not associated with the empiric treatment of gonorrhea and chlamydia in the ED nor concordance or discordance between empiric antibiotic treatment for gonorrhea and chlamydia in the ED and resultant gonorrhea and chlamydia NAAT result.

Our study also examined whether other patient and provider demographics were associated with our outcomes of interest. We found that neither patient's age, marital status, nor the pregnancy status was associated with ≥10,000 CFU/mL of bacteriuria on urine culture in women diagnosed with UTI. We found that patient encounters principally involving a resident plus attending, compared with encounters involving APPs, were more likely to have ≥10,000 CFU/mL on urine culture in patients diagnosed with a UTI. These findings may reflect selection bias as the APPs tend to care for less severely ill patients in the ED compared with residents.

Black/African American women are significantly more likely to test positive for gonorrhea, chlamydia, and trichomonas than other races [29]. However, when accounting for other triage, demographic, and laboratory factors, patient's race was not significantly associated with higher rates of empiric antibiotic treatment for gonorrhea and chlamydia in the ED. Patient's race was also not associated with discordance between empiric antibiotics for gonorrhea and chlamydia administered in the ED and the NAAT testing results. Previously, it was shown that non-Hispanic race and ethnicity, treatment by a nurse practitioner, and older age were associated with the empiric treatment of STIs [33]. In addition, the empiric treatment of STIs in a pediatric ED was more common among non-Hispanic White patients than among non-Hispanic Black patients despite lower disease prevalence in the non-Hispanic White population [33]. A survey of primary care physicians showed that screening for STIs in asymptomatic adolescent women was significantly more likely if the practitioner was a woman and the practice had more Black/African American patients [34]. Other studies have shown that race was not a significant predictor of undertreatment for STIs, but Black patients were significantly more likely to be overtreated and to receive nonrecommended treatments for STIs than White patients [35, 36].

Younger age, being single (compared to married/life partner), and having higher vaginal WBCs on the wet mount were significantly associated with receiving empiric antibiotics for gonorrhea and chlamydia, which have also been reported previously [24, 27, 29, 37, 38]. ED clinicians were more likely to empirically treat gonorrhea and chlamydia in the ED when trichomonas was identified during the ED encounter. Patient encounters involving an APP (vs. attending only) were significantly more likely to treat for gonorrhea and chlamydia empirically, which may reflect the lower acuity patients that the APPs were more likely to encounter in a “fast track” area of the ED. However, our regression models did account for ESI. The sex of the primary ED provider was not associated with being more likely to receive empiric antibiotics for gonorrhea and chlamydia.

While race was not associated with discordance between being given empiric antibiotics for gonorrhea and chlamydia and the eventual NAAT results, the following demographics were younger age, being single (vs. married/life partner), and being treated in the ED by an APP (vs. attending). These associations are likely because there was approximately five times the number of patients empirically treated for gonorrhea and chlamydia who tested negative compared to those who tested positive for one or both of those infections. There may have been social factors that required empiric antibiotics for gonorrhea and chlamydia in the ED in some of these encounters. However, our findings highlight an opportunity for improved antibiotics stewardship and shared decision making with the patient about the appropriateness of empiric treatment for STIs.

## 5. Limitations

Our data set only included patients tested for an STI or who received a urinalysis and urine culture. We missed patients diagnosed with a UTI based on urinalysis only and who did not get a urine culture. We did not examine patients empirically treated for STIs but who did not undergo STI testing. Our data set did not include history or physical exam findings which could have been included in our regression models; however, the history and physical examination findings may be unreliable for predicting STIs [27, 39]. We recognize that UTIs are frequently mismanaged and that ICD codes are an imperfect strategy for identifying disease. However, our UTI outcome focused on the diagnosis the ED provider thought was accurate at the time of the ED encounter. In addition, we could not differentiate complicated from uncomplicated UTIs or whether some patients had multiple ED encounters, which could have influenced prescriber decisions on ordering a urine culture or testing and treating for an STI. Our data set comprised data from a single healthcare system, so the results may not be generalizable. In addition, the data were collected years ago, so more current data could influence the results.

We only examined women from a single health system who were not admitted to the hospital, so our results are not generalizable to all demographics and all ED encounters. While we only examined bacterial urine cultures, viruses, fungi, and some noninfectious conditions can also cause UTI symptoms. We used 10,000 CFU/mL as our cutoff value, but no consensus exists for a specific CFU/mL that presents all UTIs.

Our data set had limited racial diversity, which was incompletely dichotomized. Adding other known social determinants of health could have improved our models.

Alternative antibiotics to the ones chosen in our models could have been used to treat gonorrhea and chlamydia in the ED. The U.S. Centers for Disease Control and Prevention updated STI testing and treatment recommendations in 2021, which occurred after the patient encounters in our data set [29]. We did not have information on the dose or duration of the antibiotics reported in the study, so some patients may have been treated with ceftriaxone plus azithromycin or doxycycline for a non-STI indication, but this is very unlikely in the setting of concurrent STI testing. We did not account for ED diagnoses that could also have affected our results, such as pelvic inflammatory disease (PID), which may be treated by antibiotics with activity against gonorrhea and chlamydia even though non-STI bacteria may have caused the condition. Regardless, patients with PID routinely get tested for STIs in the ED.

History or physical examination findings could have influenced the provider's decision to treat STIs in the ED. However, the history and physical examination may be less important for correctly diagnosing gonorrhea and chlamydia than demographic and laboratory findings [27, 39]. Information on a patient's sexual orientation, number of sexual partners, and the sex of sexual partners could have influenced STI treatment decisions. However, ED providers have historically done poorly in taking detailed sexual histories or adhering to STI testing and treatment guidelines [39, 40]. Other demographic variables not included in our data set could have affected the decision to treat STIs in the ED, such as homelessness or limited means to return to the ED for treatment if STI testing was positive. Poverty and lower socioeconomic status are both associated with higher rates of STIs [41, 42]. We did not include male patients in our analysis because those with penile discharge may be more likely to undergo preemptive STI treatment without concurrent testing. Therefore, they would not be included in our data set.

## 6. Conclusion

We did not observe significant differences between patient's race and the presence or absence of bacteriuria among patients diagnosed with a UTI or in managing gonorrhea and chlamydia infections. The patient's age and marital status, but not the provider's sex, were significantly associated with differences in the management of gonorrhea and chlamydia.

## Figures and Tables

**Table 1:** Descriptive analysis of patient demographics and clinical characteristics for all encounters by patient's race.

	White (*N* = 11,995)	Asian/other race (*N* = 638)	Black/African American (*N* = 25,429)	Total (*N* = 38,062)
Age (years)				
18–28	2,845 (23.7%)	277 (43.4%)	13,244 (52.1%)	16,366 (43.0%)
29–39	2,025 (16.9%)	165 (25.9%)	5,898 (23.2%)	8,088 (21.2%)
40–50	1,434 (12.0%)	80 (12.5%)	2,484 (9.8%)	3,998 (10.5%)
51–61	1,343 (11.2%)	57 (8.9%)	1,446 (5.7%)	2,846 (7.5%)
≥62	4,348 (36.2%)	59 (9.2%)	2,357 (9.3%)	6,764 (17.8%)
Marital status				
Missing	45	8	47	100
Single	4,320 (36.2%)	333 (52.9%)	20,603 (81.2%)	25,256 (66.5%)
Married/life partner	4,102 (34.3%)	247 (39.2%)	2,546 (10.0%)	6,895 (18.2%)
Separated/divorced	1,448 (12.1%)	25 (4.0%)	1,257 (5.0%)	2,730 (7.2%)
Widowed	2,080 (17.4%)	25 (4.0%)	976 (3.8%)	3,081 (8.1%)
Documented primary care physician				
No	6,576 (54.8%)	469 (73.5%)	19,949 (78.4%)	26,994 (70.9%)
Yes	5,419 (45.2%)	169 (26.5%)	5,480 (21.6%)	11,068 (29.1%)
Pregnant				
No	11, 406 (95.1%)	543 (85.1%)	21,421 (84.2%)	33,370 (87.7%)
Yes	589 (4.9%)	95 (14.9%)	4,008 (15.8%)	4,692 (12.3%)
Emergency severity index				
Missing	207	32	1,189	1,428
1 and 2	1,063 (9.0%)	46 (7.6%)	1,104 (4.6%)	2,213 (6.0%)
3	9,111 (77.3%)	448 (73.9%)	17,996 (74.2%)	27,555 (75.2%)
4 and 5	1,614 (13.7%)	112 (18.5%)	5,140 (21.2%)	6,866 (18.7%)
Mechanism of ED arrival				
Missing	112	4	131	247
Private vehicle	9,194 (77.4%)	534 (84.2%)	21,034 (83.1%)	30,762 (81.3%)
EMS/Police	2,596 (21.8%)	81 (12.8%)	3,153 (12.5%)	5,830 (15.4%)
Public transport/on foot	93 (0.8%)	19 (3.0%)	1,111 (4.4%)	1,223 (3.2%)
Sex of primary ED provider				
Missing	741	24	203	968
Female	3,469 (30.8%)	194 (31.6%)	8,228 (32.6%)	11,891 (32.1%)
Male	7,785 (69.2%)	420 (68.4%)	16,998 (67.4%)	25,203 (67.9%)
Training level of primary ED provider				
Missing	84	13	348	445
Attending only	7690 (64.6%)	338 (54.1%)	11282 (45.0%)	19310 (51.3%)
APP	2499 (21.0%)	119 (19.0%)	5335 (21.3%)	7953 (21.1%)
Attending + Resident	1722 (14.5%)	168 (26.9%)	8464 (33.7%)	10354 (27.5%)
Urinalysis performed				
Not performed	748 (6.2%)	56 (8.8%)	2,716 (10.7%)	3,520 (9.2%)
Performed	11,247 (93.8%)	582 (91.2%)	22,713 (89.3%)	34,542 (90.8%)
Source of urine sample				
Missing	728	56	2,653	3,437
Clean catch/void urine	6616 (58.7%)	309 (53.1%)	8,838 (38.8%)	15,763 (45.5%)
Not documented	3,349 (29.7%)	249 (42.8%)	13,259 (58.2%)	16,857 (48.7%)
Other	1,302 (11.6%)	24 (4.1%)	679 (3.0%)	2,005 (5.8%)
Bacteria, urine (0–4+)				
Missing	2,882	178	8,145	11,205
Median (Q1, Q3)	1.0 (0.0, 2.0)	1.0 (0.0, 2.0)	1.0 (0.0, 2.0)	1.0 (0.0, 2.0)
Blood, urine (0–3+)				
Missing	985	67	3,177	4,229
Median (Q1, Q3)	0.0 (0.0, 2.0)	0.0 (0.0, 2.0)	0.0 (0.0, 1.0)	0.0 (0.0, 2.0)
Glucose, urine				
Missing	809	56	2,856	3,721
Negative	10,369 (92.7%)	536 (92.1%)	21,256 (94.2%)	32,161 (93.7%)
Positive	817 (7.3%)	46 (7.9%)	1,317 (5.8%)	2,180 (6.3%)
Ketones, urine				
Missing	818	56	2,882	3,756
Negative	9,339 (83.6%)	483 (83.0%)	18,475 (81.9%)	28,297 (82.5%)
Positive	1,838 (16.4%)	99 (17.0%)	4,072 (18.1%)	6,009 (17.5%)
Leukocyte esterase, urine (0–3+)				
Missing	1,049	66	3,185	4,300
Median (Q1, Q3)	1.0 (0.0, 3.0)	1.0 (0.0, 2.0)	1.0 (0.0, 2.0)	1.0 (0.0, 2.0)
Mucous, urine (0–4+)				
Missing	2,905	178	8,142	11,225
Median (Q1, Q3)	0.0 (0.0, 1.0)	0.0 (0.0, 1.0)	1.0 (0.0, 2.0)	0.0 (0.0, 1.0)
Nitrite, urine				
Missing	805	56	2,826	3,687
Negative	10,020 (89.5%)	546 (93.8%)	21,102 (93.4%)	31,668 (92.1%)
Positive	1,170 (10.5%)	36 (6.2%)	1,501 (6.6%)	2,707 (7.9%)
Protein, urine				
Missing	820	57	2,867	3,744
Negative	6,878 (61.5%)	383 (65.9%)	14,734 (65.3%)	21,995 (64.1%)
Positive	4,297 (38.5%)	198 (34.1%)	7,828 (34.7%)	12,323 (35.9%)
RBCs, urine (0–101)				
Missing	2,910	180	8,136	11,226
Median (Q1, Q3)	2.5 (2.5, 12.5)	2.5 (2.5, 12.5)	2.5 (2.0, 12.5)	2.5 (2.5, 12.5)
Trichomonas, urine				
Missing	2,917	178	8,209	11,304
Not present	9033 (99.5%)	456 (99.1%)	16886 (98.1%)	26375 (98.6%)
Present	45 (0.5%)	4 (0.9%)	334 (1.9%)	383 (1.4%)
WBC clumps, urine				
Missing	3,026	182	8332	11,540
None	8, 121 (90.5%)	425 (93.2%)	15,817 (92.5%)	24,363 (91.9%)
Present	848 (9.5%)	31 (6.8%)	1,280 (7.5%)	2,159 (8.1%)
WBC, urine (0–101)				
Missing	2,969	180	8,120	11,269
Median (Q1, Q3)	12.5 (2.5, 36.0)	8.0 (2.5, 24.8)	8.0 (2.5, 29.0)	12.5 (2.5, 36.0)
Yeast, urine				
Missing	3,025	180	8,238	11,443
None	8,635 (96.3%)	446 (97.4%)	16,706 (97.2%)	25,787 (96.9%)
Present	335 (3.7%)	12 (2.6%)	485 (2.8%)	832 (3.1%)
Clue cells, wet mount				
No result	10,577 (88.2%)	426 (66.8%)	11,358 (44.7%)	22,361 (58.7%)
None	1,038 (8.7%)	152 (23.8%)	7,603 (29.9%)	8,793 (23.1%)
Present	380 (3.2%)	60 (9.4%)	6,468 (25.4%)	6,908 (18.1%)
WBC, wet mount				
No result	10,509 (87.6%)	423 (66.3%)	11,032 (43.4%)	21,964 (57.7%)
≤10	1,050 (8.8%)	147 (23.0%)	9,632 (37.9%)	10,829 (28.5%)
11–100	436 (3.6%)	68 (10.7%)	4,765 (18.7%)	5,269 (13.8%)
Yeast, wet mount				
No result	10,587 (88.3%)	426 (66.8%)	11,549 (45.4%)	22,562 (59.3%)
None	1,355 (11.3%)	195 (30.6%)	12,925 (50.8%)	14,475 (38.0%)
Present	53 (0.4%)	17 (2.7%)	955 (3.8%)	1,025 (2.7%)
Trichomonas, wet mount				
No result	10,588 (88.3%)	428 (67.1%)	11,539 (45.4%)	22,555 (59.3%)
None	1,361 (11.3%)	203 (31.8%)	12,596 (49.5%)	14,160 (37.2%)
Present	46 (0.4%)	7 (1.1%)	1,294 (5.1%)	1,347 (3.5%)
Gonorrhea, NAAT				
No test result	10,489 (87.4%)	423 (66.3%)	10,923 (43.0%)	21,835 (57.4%)
Negative	1,489 (12.4%)	211 (33.1%)	14,051 (55.3%)	15,751 (41.4%)
Positive	17 (0.1%)	4 (0.6%)	455 (1.8%)	476 (1.3%)
Chlamydia, NAAAT				
No test result	10,489 (87.4%)	422 (66.1%)	10,931 (43.0%)	21,842 (57.4%)
Negative	1,426 (11.9%)	203 (31.8%)	13,291 (52.3%)	14,920 (39.2%)
Positive	80 (0.7%)	13 (2.0%)	1,207 (4.7%)	1,300 (3.4%)
Trichomonas, NAAT				
No test result	11,539 (96.2%)	569 (89.2%)	21,053 (82.8%)	33,161 (87.1%)
Negative	439 (3.7%)	66 (10.3%)	3,978 (15.6%)	4,483 (11.8%)
Positive	17 (0.1%)	3 (0.5%)	398 (1.6%)	418 (1.1%)
Diagnosed with a urinary tract infection (UTI in the ED)				
No	8,384 (69.9%)	496 (77.7%)	20,895 (82.2%)	29,775 (78.2%)
Yes	3,611 (30.1%)	142 (22.3%)	4,534 (17.8%)	8,287 (21.8%)

^^^There were 192 encounters with no race provided.

**Table 2:** Urine culture results among women diagnosed with a UTI in the ED.

	White (*N* = 3,611)	Asian/other race (*N* = 142)	Black/African American (*N* = 4,534)	Total (*N* = 8,287)	*p* value
Colony forming units (CFU)/mL of bacteria in urine culture (binary)					<0.001 [1]
(i) <10,000	1,857 (51.4%)	70 (49.3%)	1,967 (43.4%)	3,894 (47.0%)	
(ii) ≥10,000	1,355 (37.5%)	53 (37.3%)	1,552 (34.2%)	2,960 (35.7%)	
(iii) No urine culture	399 (11.0%)	19 (13.4%)	1,015 (22.4%)	1,433 (17.3%)	

**Table 3:** Empiric antibiotic treatment for gonorrhea and chlamydia during the ED encounter and gonorrhea and chlamydia testing result.

	White (*N* = 1,503)	Asian/other race (*N* = 214)	Black/African American (*N* = 14,482)	Total (*N* = 16,199)
Negative for both gonorrhea and chlamydia, and no empiric antibiotics given for gonorrhea and chlamydia during ED encounter (concordance)	1,237 (10.3%)	171 (1.4%)	10,610 (88.3%)	12,018
Negative test for both gonorrhea and chlamydia, and empiric antibiotics given for gonorrhea and chlamydia during ED encounter (discordance)	176 (6.9%)	28 (1.1%)	2,356 (92.0%)	2,560
Positive test for gonorrhea, chlamydia, or both, and no empiric antibiotics given for gonorrhea and chlamydia during ED encounter (discordance)	62 (5.6%)	11 (1.0%)	1,040 (93.4%)	1,113
Positive test for gonorrhea, chlamydia, or both, and empiric antibiotics given for gonorrhea and chlamydia during ED encounter (concordance)	28 (5.5%)	4 (0.8%)	476 (93.7%)	508

**Table 4:** Multivariate OR of being diagnosed with a UTI and having a positive urine culture (vs. negative urine culture).

Variables	Comparison groups	Reference levels	OR (95% CI)	*p* value
Age (years)	18–28	≥62	0.93 (0.74, 1.18)	0.56
	29–39	≥62	0.89 (0.71, 1.13)	0.35
	40–50	≥62	0.86 (0.67, 1.10)	0.22
	51–61	≥62	0.94 (0.74, 1.20)	0.64

Race	Black/African American	Asian/other	1.09 (0.69, 1.73)	0.70
	White	Asian/other	0.84 (0.53, 1.33)	0.44

Marital status	Separated/divorced	Married/life partner	0.88 (0.69, 1.12)	0.31
	Single	Married/life partner	1.01 (0.84, 1.21)	0.91
	Widowed	Married/life partner	1.10 (0.87, 1.38)	0.42

Documented primary care physician	Yes	No	0.89 (0.78, 1.02)	0.08

Pregnant	Yes	No	0.80 (0.57, 1.13)	0.21

Emergency severity index	3	1-2	1.04 (0.79, 1.40)	0.77
	4-5	1-2	1.44 (1.05, 1.98)	0.02

Mechanism of ED arrival	Private vehicle	EMS/police	1.19 (0.98, 1.45)	0.09
	Public transport/on foot	EMS/police	1.52 (0.95, 2.41)	0.08

Sex of primary ED provider	Male	Female	1.0257 (0.89, 1.18)	0.72

Training level of primary ED provider	Attending only	APP	1.08 (0.92, 1.26)	0.35
	Attending plus resident	APP	1.27 (1.05, 1.54)	0.02

Source of urine sample	Not documented	Clean catch/voided	0.62 (0.54, 0.72)	<0.001
	Other	Clean catch/voided	1.38 (1.08, 1.78)	0.01

Bacteria (urinalysis)	1-unit increase		1.31 (1.25, 1.38)	<0.001

Blood (urinalysis)	1-unit increase		1.24 (1.15, 1.32)	<0.001

Glucose (urinalysis)	Positive	Negative	0.97 (0.77, 1.23)	0.80

Ketones (urinalysis)	Positive	Negative	0.82 (0.68, 0.98)	0.03

Leukocyte esterase (urinalysis)	1-unit increase		0.99 (0.93, 1.07)	0.89

Mucous (urinalysis)	1-unit increase		0.89 (0.84, 0.95)	<0.001

Nitrite (urinalysis)	Positive	Negative	6.42 (5.40, 7.67)	<0.001

Protein (urinalysis)	Positive	Negative	1.09 (0.95, 1.25)	0.23

RBCs (urinalysis)	1-unit increase		1.00 (1.00, 1.00)	0.12

WBC clumps (urinalysis)	Present	Negative	1.50 (1.26, 1.78)	<0.001

WBCs (urinalysis)	1-unit increase		1.02 (1.01, 1.02)	<0.001

Yeast (urinalysis)	Present	Absent	0.74 (0.53, 1.03)	0.08

Clue cells (vaginal wet mount)	Not performed	Negative	0.32 (0.02, 3.58)	0.37
	Present	Negative	0.81 (0.56, 1.16)	0.25

WBC (vaginal wet mount) (cells/HPF)	11–100	≤10	0.58 (0.40, 0.82)	0.002
	Not performed	≤10	1.29 (0.28, 8.19)	0.77

Yeast (vaginal wet mount)	Not performed	Negative	1.11 (0.13, 9.70)	0.93
	Present	Negative	0.78 (0.37, 1.56)	0.50

*T. vaginalis* NAAT	No test	Negative	0.61 (0.42, 0.90)	0.01
	Positive	Negative	0.60 (0.21, 1.55)	0.31

Positive for gonorrhea, chlamydia, or both; negative for both gonorrhea and chlamydia; or not tested for gonorrhea and chlamydia	Not tested for gonorrhea and chlamydia	Negative for both gonorrhea and chlamydia	1.03 (0.59, 1.77)	0.93
	Positive for gonorrhea, chlamydia, or both	Negative for both gonorrhea and chlamydia	1.06 (0.65, 1.73)	0.81

Trichomonas found during the ED encounter	Positive for *T. vaginalis* during the ED encounter	Negative for *T. vaginalis* infection during ED encounter	0.41 (0.20, 0.78)	0.009
	Vaginal wet mount not performed	Negative for *T. vaginalis* infection during ED encounter	2.16 (0.69, 7.31)	0.20

**Table 5:** Multivariable logistic regression for associations between empiric treatment for gonorrhea and chlamydia (vs. no treatment in the ED) among those tested.

Variables	Comparison groups	Reference levels	OR (95% CI)	*p* value
Age (years)	18–28	≥62	3.96 (1.51, 13.65)	0.01
	29–39	≥62	3.81 (1.45, 13.15)	0.01
	40–50	≥62	3.86 (1.45, 13.40)	0.01
	51–61	≥62	4.40 (1.60, 15.65)	0.009

Race	Black/African American	Asian/other	1.85 (1.00, 3.75)	0.07
	White	Asian/other	1.598 (0.84, 3.32)	0.18

Marital status	Separated/divorced	Married/life partner	1.49 (1.02, 2.18)	0.04
	Single	Married/life partner	1.60 (1.26, 2.06)	<0.001
	Widowed	Married/life partner	0.71 (0.16, 2.20)	0.59

Documented primary care physician	Yes	No	0.89 (0.76, 1.04)	0.14

Pregnant	Yes	No	0.16 (0.12, 0.19)	<0.001

Emergency severity index	3	1-2	0.95 (0.63, 1.46)	0.80
	4-5	1-2	1.07 (0.70, 1.66)	0.77

Mechanism of ED arrival	Private vehicle	EMS/police	1.10 (0.87, 1.42)	0.42
	Public transport/on foot	EMS/police	1.06 (0.75, 1.49)	0.73

Sex of primary ED provider	Male	Female	1.10 (0.98, 1.25)	0.12

Training level of primary ED provider	Attending only	APP	0.76 (0.65, 0.89)	<0.001
	Attending plus resident	APP	0.99 (0.85, 1.16)	0.94

Source of urine sample	Not documented	Clean catch/voided	0.72 (0.56, 0.91)	0.007
	Other	Clean catch/voided	0.77 (0.27, 1.84)	0.58

Bacteria (urinalysis)	1-unit increase		1.02 (0.97, 1.08)	0.43

Blood (urinalysis)	1-unit increase		0.84 (0.79, 0.89)	<0.001

Glucose (urinalysis)	Positive	Negative	0.86 (0.63, 1.15)	0.32

Ketones (urinalysis)	Positive	Negative	1.05 (0.90, 1.23)	0.54

Leukocyte esterase (urinalysis)	1-Unit increase		1.17 (1.10, 1.25)	<0.001

Mucous (urinalysis)	1-Unit increase		1.00 (0.96, 1.05)	0.83

Nitrite (urinalysis)	Positive	Negative	1.06 (0.80, 1.39)	0.68

Protein (urinalysis)	Positive	Negative	0.95 (0.83, 1.08)	0.45

RBCs (urinalysis)	1-unit increase		0.99 (0.99, 1.00)	<0.001

WBC clumps (urinalysis)	Present	Negative	1.19 (0.90, 1.56)	0.21

WBCs (urinalysis)	1-unit increase		1.00 (1.00, 1.01)	0.12

Yeast (urinalysis)	Present	Absent	0.43 (0.26, 0.66)	<0.001

CFU/mL on urine culture	≥10,000	<10,000	0.86 (0.66, 1.11)	0.25
	No urine culture done	<10,000	1.24 (0.99, 1.57)	0.07

Clue cells (vaginal wet mount)	Not performed	Negative	0.35 (0.12, 0.97)	0.05
	Present	Negative	0.98 (0.87, 1.10)	0.74

WBC (vaginal wet mount) (cells/HPF)	11–100	≤10	1.38 (1.22, 1.56)	<0.001
	Not performed	≤10	1.09 (0.51, 2.48)	0.83

Yeast (vaginal wet mount)	Not performed	Negative	2.01 (0.69, 6.27)	0.21
	Present	Negative	0.79 (0.62, 1.01)	0.06

*N. gonorrhea* NAAT	No test result	Negative	3.70 (0.97, 13.81)	0.048
	Positive	Negative	1.95 (1.50, 2.52)	<0.001

*C. trichomonas* NAAT	No test result	Negative	1.55 (0.46, 4.50)	0.44
	Positive	Negative	1.49 (1.24, 1.78)	<0.001

*T. vaginalis* NAAT	No test result	Negative	1.10 (0.95, 1.27)	0.22
	Positive	Negative	1.25 (0.89, 1.74)	0.19

Diagnosed with a UTI in the ED	Yes	No	0.76 (0.65, 0.90)	0.001

Trichomonas found during the ED encounter	Positive for *T. vaginalis* during the ED encounter	Negative for *T. vaginalis* infection during ED encounter	3.88 (3.29, 4.58)	<0.001
	Vaginal wet mount not performed	Negative for *T. vaginalis* infection during ED encounter	1.27 (0.49, 3.20)	0.62

**Table 6:** Multivariable logistic regression for associations between discordance between gonorrhea and chlamydia test results and empiric ED treatment (vs. concordance).

Variables	Comparison groups	Reference levels	OR (95% CI)	*p* value
Age (years)	18–28	≥62	0.19 (0.06, 0.49)	0.002
	29–39	≥62	0.25 (0.07, 0.65)	0.01
	40–50	≥62	0.27 (0.08, 0.71)	0.02
	51–61	≥62	0.24 (0.07, 0.65)	0.01

Race	Black/African American	Asian/other	0.59 (0.32, 1.00)	0.06
	White	Asian/other	0.75 (0.40, 1.31)	0.33

Marital status	Separated/divorced	Married/life partner	0.57 (0.41, 0.81)	0.002
	Single	Married/life partner	0.65 (0.52, 0.81)	<0.001
	Widowed	Married/life partner	2.5 (0.70, 16.30)	0.23

Documented primary care physician	Yes	No	1.09 (0.95, 1.25)	0.24

Pregnant	Yes	No	2.66 (2.30, 3.10)	<0.001

Emergency severity index	3	1-2	0.96 (0.65, 1.39)	0.84
	4-5	1-2	0.94 (0.63, 1.38)	0.77

Mechanism of ED arrival	Private vehicle	EMS/police	1.08 (0.88, 1.33)	0.45
	Public transport/on foot	EMS/police	1.11 (0.83, 1.50)	0.48

Sex of primary ED provider	Male	Female	0.99 (0.88, 1.10)	0.82

Training level of primary ED provider	Attending only	APP	1.05 (0.91, 1.20)	0.53
	Attending plus resident	APP	0.91 (0.79, 1.05)	0.21

Source of urine sample	Not documented	Clean catch/voided	1.29 (1.04, 1.61)	0.02
	Other	Clean catch/voided	1.27 (0.57, 3.24)	0.58

Bacteria (urinalysis)	1-unit increase		1.04 (0.99, 1.09)	0.16

Blood (urinalysis)	1-unit increase		1.15 (1.09, 1.22)	<0.001

Glucose (urinalysis)	Positive	Negative	1.27 (0.97, 1.69)	0.09

Ketones (urinalysis)	Positive	Negative	1.04 (0.91, 1.20)	0.57

Leukocyte esterase (urinalysis)	1-unit increase		0.84 (0.79, 0.89)	<0.001

Mucous (urinalysis)	1-unit increase		1.00 (0.96, 1.04)	0.98

Nitrite (urinalysis)	Positive	Negative	0.85 (0.67, 1.09)	0.19

Protein (urinalysis)	Positive	Negative	0.98 (0.87, 1.10)	0.77

RBCs (urinalysis)	1-unit increase		1.00 (1.00, 1.00)	0.19

WBC clumps (urinalysis)	Present	Negative	0.91 (0.71, 1.17)	0.47

WBCs (urinalysis)	1-unit increase		1.00 (0.9964, 1.0016)	0.46

Yeast (urinalysis)	Present	Absent	1.77 (1.22, 2.63)	0.004

CFU/mL on urine culture	≥10,000	<10,000	1.11 (0.88, 1.40)	0.38
	No urine culture done	<10,000	0.77 (0.62, 0.94)	0.01

Clue cells (vaginal wet mount)	Not performed	Negative	1.86 (0.75, 4.64)	0.18
	Present	Negative	0.83 (0.75, 0.93)	<0.001

WBC (vaginal wet mount) (cells/HPF)	11–100	≤10	0.71 (0.64, 0.79)	<0.001
	Not performed	≤10	1.00 (0.50, 1.91)	>0.99

Yeast (vaginal wet mount)	Not performed	Negative	0.77 (0.28, 2.04)	0.60
	Present	Negative	1.38 (1.11, 1.7)	0.004

*T. vaginalis* NAAT	No test result	Negative	1.02 (0.90, 1.16)	0.78
	Positive	Negative	0.60 (0.45, 0.80)	<0.001

Diagnosed with a UTI in the ED	Yes	No	1.11 (0.96, 1.29)	0.16

Trichomonas found during the ED encounter	Vaginal wet mount not performed	Negative for *T. vaginalis* infection during ED encounter	1.55 (0.64, 3.79)	0.33
	Positive for *T. vaginalis* during the ED encounter	Negative for *T. vaginalis* infection during ED encounter	0.63 (0.26, 1.52)	0.30

## Data Availability

The dataset is not available for dissemination.

## Supplementary Materials

Supplementary Materials Supplementary file 1: the univariate analysis for UTI diagnosis and ≥10,000 CFU/mL bacteriuria vs. <10,000 CFU/mL bacteriuria on urine culture. Supplementary file 2: the univariate analysis for the empiric treatment for gonorrhea and chlamydia in those tested for the diseases.
