# Supplementary material for: Patient and Provider Demographics and the Management of Genitourinary Tract Infections in the Emergency Department
Source: Emerg Med Int. 2023 Sep 11;2023:1522347. doi: 10.1155/2023/1522347 (PMC10506883; doi:10.1155/2023/1522347)
Supplement: Supplementary Materials — Supplementary file 1: the univariate analysis for UTI diagnosis and ≥10,000 CFU/mL bacteriuria vs. <10,000 CFU/mL bacteriuria on urine culture. Supplementary file 2: the univariate analysis for the empiric treatment for gonorrhea and chlamydia in those tested for the diseases. [file 1522347.f1.zip › Supplement 1 (1).docx]

### Supplement 1. Univariate analysis of women diagnosed with a UTI and having a urine culture ≥10,000 CFU/mL (vs <10,000 CFU/mL)

| Variable | OR(95% CI) | P Value | Overall P Value |
| --- | --- | --- | --- |
| Age 29-39 years (vs. Age 18-28) | 0.98 (0.85, 1.13) | 0.80 | 0.02 |
| Age 40-50 years (vs. Age 18-28) | 0.99 (0.84, 1.17) | 0.93 |  |
| Age 51-61 years(vs. Age 18-28) | 1.10 (0.93, 1.31) | 0.27 |  |
| Age ≥62 years (vs. Age 18-28) | 1.20 (1.06, 1.36) | 0.003 |  |
| Race Asian/Other race (vs. White) | 1.04 (0.72, 1.49) | 0.84 | 0.28 |
| Race Black/African American (vs. White) | 1.08 (0.98, 1.19) | 0.11 |  |
| Marital status Married/Life Partner (vs. Single) | 1.03 (0.92, 1.16) | 0.59 | 0.08 |
| Marital status Separated/Divorced (vs. Single) | 1.05 (0.88, 1.24) | 0.61 |  |
| Marital status Widowed (vs. Single) | 1.21 (1.05, 1.40) | 0.01 |  |
| Documented primary care physician (vs. not documented/none) | 0.96 (0.87, 1.06) | 0.44 | 0.44 |
| Pregnant (vs. not) | 0.47 (0.36, 0.61) | < .001 | <.001 |
| Emergency severity index (ESI) 3 (vs. ESI 1-2) | 0.97 (0.77, 1.22) | 0.77 | <.001 |
| Emergency severity index (ESI) 4 and 5 (vs. ESI 1-2) | 1.39 (1.1, 1.77) | 0.007 |  |
| Method of ED arrival EMS/Police (vs. Private vehicle) | 1.21 (1.06, 1.38) | 0.004 | 0.01 |
| Method of ED arrival Public Transport/On Foot (vs. Private vehicle) | 1.19 (0.86, 1.63) | 0.30 |  |
| Sex of primary ED provider Male (vs. Female) | 1.02 (0.92, 1.14) | 0.68 | 0.68 |
| Training level of primary ED provider APP (vs. Attending only) | 0.92 (0.82, 1.04) | 0.19 | 0.04 |
| Training level of primary ED provider Attending+Resident (vs. Attending only) | 1.12 (0.99, 1.27) | 0.08 |  |
| Bacteria, Urine | 1.47 (1.41, 1.52) | < .001 | < .001 |
| Blood, Urine | 1.40 (1.34, 1.46) | < .001 | < .001 |
| Glucose, Urine (vs. none) | 1.04 (0.87, 1.25) | 0.65 | 0.65 |
| Ketones, Urine (vs. none) | 0.74 (0.65, 0.85) | < .001 | < .001 |
| Leukocyte Esterase, Urine | 1.30 (1.25, 1.37) | < .001 | < .001 |
| Mucous, Urine | 0.87 (0.84, 0.91) | < .001 | < .001 |
| Nitrite, Urine Positive (vs. negative) | 6.81 (5.93, 7.85) | < .001 | < .001 |
| Protein, Urine Positive (vs. negative) | 1.81 (1.64, 1.99) | < .001 | < .001 |
| RBCs, Urine | 1.01 (1.01, 1.01) | < .001 | < .001 |
| Trichomonas, Urine Present (vs. negative) | 0.17 (0.07, 0.33) | < .001 | < .001 |
| WBC Clumps, Urine Present (vs. not present) | 2.58 (2.25, 2.95) | < .001 | < .001 |
| WBC, Urine | 1.02 (1.02, 1.02) | < .001 | < .001 |
| Yeast, Urine (vs. none) | 0.80 (0.62, 1.04) | 0.10 | 0.10 |
| Clue cells, wet mount None (vs. not performed) | 0.75 (0.63, 0.90) | 0.002 | < .001 |
| Clue cells, wet mount Present (vs. not performed) | 0.64 (0.51, 0.81) | < .001 |  |
| WBC, wet mount ≤10 cells/HPF (vs. not performed) | 0.87 (0.73, 1.04) | 0.13 | < .001 |
| WBC, wet mount 11-100 cells/HPF (vs. not performed) | 0.49 (0.39, 0.61) | < .001 |  |
| Yeast, wet mount None (vs. not performed) | 0.75 (0.65, 0.88) | < .001 | < .001 |
| Yeast, wet mount Present (vs. not performed) | 0.40 (0.22, 0.69) | 0.002 |  |
| Trichomonas, wet mount None (vs. not performed) | 0.78 (0.67, 0.91) | 0.001 | < .001 |
| Trichomonas, wet mount Present (vs. not performed) | 0.34 (0.21, 0.55) | < .001 |  |
| Gonorrhea NAAT Negative (vs. not performed) | 0.71 (0.61, 0.82) | < .001 | < .001 |
| Gonorrhea NAAT Positive (vs. not performed) | 0.57 (0.3, 1.03) | 0.07 |  |
| Chlamydia NAAT Negative (vs. not performed) | 0.69 (0.59, 0.80) | < .001 | < .001 |
| Chlamydia NAAT Positive (vs. not performed) | 0.86 (0.58, 1.28) | 0.47 |  |
| Trichomonas NAAT Negative (vs. not performed) | 0.99 (0.77, 1.28) | 0.94 | 0.07 |
| Trichomonas NAAT Positive (vs. not performed) | 0.44 (0.19, 0.89) | 0.03 |  |
