# Supplementary material for: Patient and Provider Demographics and the Management of Genitourinary Tract Infections in the Emergency Department
Source: Emerg Med Int. 2023 Sep 11;2023:1522347. doi: 10.1155/2023/1522347 (PMC10506883; doi:10.1155/2023/1522347)
Supplement: Supplementary Materials — Supplementary file 1: the univariate analysis for UTI diagnosis and ≥10,000 CFU/mL bacteriuria vs. <10,000 CFU/mL bacteriuria on urine culture. Supplementary file 2: the univariate analysis for the empiric treatment for gonorrhea and chlamydia in those tested for the diseases. [file 1522347.f1.zip › Supplement 2 (1).docx]

Supplement 2. Univariate analysis of Empiric Antibiotics given or not for patients being tested for either Gonorrhea or Chlamydia

| Variable | OR(95% CI) | P Value | Overall P Value |
| --- | --- | --- | --- |
| Age 29-39 years (vs. Age 18-28) | 0.85 (0.78, 0.93) | < .001 | < .001 |
| Age 40-50 years (vs. Age 18-28) | 1.00 (0.86, 1.15) | 0.99 |  |
| Age 51-61 years(vs. Age 18-28) | 1.07 (0.83, 1.36) | 0.62 |  |
| Age ≥62 years (vs. Age 18-28) | 0.25 (0.11, 0.50) | < .001 |  |
| Race Asian/Other race (vs. White) | 1.10 (0.72, 1.63) | 0.64 | < .001 |
| Race Black/African American (vs. White) | 1.55 (1.33, 1.81) | < .001 |  |
| Marital status Married/Life Partner (vs. Single) | 0.48 (0.41, 0.57) | < .001 | < .001 |
| Marital status Separated/Divorced (vs. Single) | 0.91 (0.73, 1.13) | 0.41 |  |
| Marital status Widowed (vs. Single) | 0.24 (0.07, 0.57) | 0.005 |  |
| Documented primary care physician (vs. not documented/none) | 0.85 (0.77, 0.95) | 0.004 | 0.003 |
| Pregnant (vs. not) | 0.16 (0.14, 0.19) | < .001 | < .001 |
| Emergency severity index (ESI) 3 (vs. ESI 1-2) | 0.98 (0.74, 1.32) | 0.89 | < .001 |
| Emergency severity index (ESI) 4 and 5 (vs. ESI 1-2) | 1.71 (1.29, 2.31) | < .001 |  |
| Method of ED arrival EMS/Police (vs. Private vehicle) | 0.82 (0.69, 0.96) | 0.02 | 0.04 |
| Method of ED arrival Public Transport/On Foot (vs. Private vehicle) | 0.93 (0.77, 1.11) | 0.41 |  |
| Sex of primary ED provider Male (vs. Female) | 1.10 (1.01, 1.20) | 0.02 | 0.02 |
| Training level of primary ED provider APP (vs. Attending only) | 1.23 (1.11, 1.36) | < .001 | < .001 |
| Training level of primary ED provider Attending+Resident (vs. Attending only) | 1.14 (1.04, 1.25) | 0.004 |  |
| Bacteria, Urine | 1.10 (1.05, 1.15) | < .001 | < .001 |
| Blood, Urine | 0.79 (0.76, 0.83) | < .001 | < .001 |
| Glucose, Urine (vs. none) | 0.80 (0.63, 1.00) | 0.06 | 0.05 |
| Ketones, Urine (vs. none) | 0.88 (0.78, 0.98) | 0.02 | 0.02 |
| Leukocyte Esterase, Urine | 1.32 (1.27, 1.37) | < .001 | < .001 |
| Mucous, Urine | 1.00 (0.97, 1.04) | 0.81 | 0.81 |
| Nitrite, Urine Positive (vs. negative) | 1.23 (1.00, 1.51) | 0.05 | 0.05 |
| Protein, Urine Positive (vs. negative) | 0.92 (0.84, 1.01) | 0.10 | 0.10 |
| RBCs, Urine | 0.99 (0.99, 0.99) | < .001 | < .001 |
| Trichomonas, Urine Present (vs. negative) | 3.49 (2.73, 4.45) | < .001 | < .001 |
| WBC Clumps, Urine Present (vs. not present) | 1.56 (1.25, 1.93) | < .001 | < .001 |
| WBC, Urine | 1.01 (1.01, 1.01) | < .001 | < .001 |
| Yeast, Urine (vs. none) | 0.55 (0.37, 0.79) | 0.002 | < .001 |
| Clue cells, wet mount None (vs. not performed) | 0.87 (0.74, 1.03) | 0.11 | 0.001 |
| Clue cells, wet mount Present (vs. not performed) | 1.01 (0.85, 1.20) | 0.93 |  |
| WBC, wet mount ≤10 cells/HPF (vs. not performed) | 0.73 (0.59, 0.90) | 0.003 | < .001 |
| WBC, wet mount 11-100 cells/HPF (vs. not performed) | 1.30 (1.06, 1.61) | 0.02 |  |
| Yeast, wet mount None (vs. not performed) | 0.88 (0.76, 1.02) | 0.09 | 0.048 |
| Yeast, wet mount Present (vs. not performed) | 0.76 (0.61, 0.95) | 0.02 |  |
| Trichomonas, wet mount None (vs. not performed) | 0.79 (0.68, 0.92) | 0.002 | < .001 |
| Trichomonas, wet mount Present (vs. not performed) | 3.62 (3.02, 4.35) | < .001 |  |
| Gonorrhea NAAT Negative (vs. not performed) | 0.45 (0.19, 1.19) | 0.08 | < .001 |
| Gonorrhea NAAT Positive (vs. not performed) | 1.34 (0.55, 3.58) | 0.54 |  |
| Chlamydia NAAT Negative (vs. not performed) | 0.58 (0.27, 1.39) | 0.19 | < .001 |
| Chlamydia NAAT Positive (vs. not performed) | 1.13 (0.51, 2.72) | 0.78 |  |
| Trichomonas NAAT Negative (vs. not performed) | 0.69 (0.63, 0.76) | < .001 | < .001 |
| Trichomonas NAAT Positive (vs. not performed) | 1.19 (0.93, 1.49) | 0.16 |  |
